# Decision-Making Deficits Are Associated With Learning Impairments in Female College Students at High Risk for Anorexia Nervosa: Iowa Gambling Task and Prospect Valence Learning Model

**DOI:** 10.3389/fpsyt.2018.00759

**Published:** 2019-01-22

**Authors:** Eunchan Na, Bitna Kang, Myung-Sun Kim

**Affiliations:** ^1^Department of Psychology, Sungshin Women's University, Seoul, South Korea; ^2^Jakwang Child & Family Clinic, Seoul, South Korea

**Keywords:** decision-making, eating disorder, high risk for anorexia nervosa, Iowa gambling task, prospect valence learning model

## Abstract

This study investigated deficits in decision-making ability in female college students at high risk for anorexia nervosa (AN) using the Iowa Gambling Task (IGT) and the prospect valence learning (PVL) model. Based on scores on the Korean version of the Eating Attitude Test-26 (KEAT-26), participants were assigned to either the high risk for AN group (*n* = 42) or the control group (*n* = 43). The high risk for AN group exhibited significantly lower total net scores and block net scores on the third, fourth, and fifth blocks of the IGT than the control group did. The high risk for AN group selected cards significantly more often from the disadvantageous A and B decks and less often from the advantageous D deck than the control group did. In addition, the block net scores of the high risk for AN group did not differ across the five blocks, whereas those of the control group increased as the trials progressed. There was a significant negative correlation between IGT total net score and total score on the KEAT−26. The high risk for AN group had significantly lower values than the control group on the learning and response consistency parameters of the PVL model. These results indicate that female college students at high risk for AN have deficits in decision-making ability, and that these deficits are related to difficulties in remembering experience obtained from earlier trials and applying it to later trials. These difficulties further lead them to make decisions randomly.

## Introduction

Anorexia nervosa (AN) is characterized by distorted body image and an intense fear of gaining weight despite low actual body weight (1). Patients with AN restrict their calorie intake, exercise excessively to maintain or reduce their weight, and have rigid and stereotyped thinking or behavior regarding food and body shape (2, 3). AN has a poor prognosis (4) and the highest mortality rate among mental disorders (5). The etiologies of AN are not fully understood and evidence-based treatments for this disorder are lacking (6).

Patients with AN exhibit deficits in several cognitive domains, such as attention (7), visuospatial construction/visual memory (8, 9), and executive function (10). Deficits in decision-making ability are particularly pronounced, as patients with AN tend to prefer immediate rewards even if they incur in long-term negative consequences (11). Decision-making deficits have been found to predict the prognosis of AN (12). Additionally, poor decision-making ability is observed not only in patients with AN but also in their healthy relatives, and can therefore be regarded as a biological marker or endophenotype of AN (13).

The Iowa gambling task [IGT, (14)] is widely used to measure decision-making ability. The IGT requires selecting one deck from among four decks presented to the subject. Two decks (A and B) are disadvantageous, delivering more losses than gains, whereas the two other decks (C and D) are advantageous, delivering more gains than losses. To perform the IGT successfully, individuals should learn the expectancy values of each deck (15). Decision-making abilities are evaluated by total net score and block net scores. As trials progress, normal individuals begin to select advantageous decks more often than disadvantageous ones (16, 17).

Studies using the IGT to investigate decision-making in patients with AN have reported that these patients exhibit significantly lower total net scores and block net scores (except on the first or second block) than healthy controls (11, 18). These results indicate that patients with AN are unable to distinguish between advantageous and disadvantageous decks even as trials progress and are likely to pursue immediate gains even at the cost of greater long-term losses (19–21). Evidence from neuroimaging research gives context to the impaired IGT performance seen in patients with AN. The ventromedial prefrontal cortex, including the orbitofrontal cortex, is involved in performance on the IGT (22). Patients with AN exhibit decreased orbitofrontal cortex volume, and greater reductions in volume correspond to lower IGT total net scores in these patients (20).

However, total net scores and block net scores on the IGT cannot provide a complete explanation of the mechanisms underlying decision-making deficits. Decision-making is a complex process consisting of several components, such as the formation of preference about possible choices, selection/execution of behavior, and evaluation of the behavior's probable outcomes (23). Several cognitive models have been developed to identify the mechanisms of processes involved in IGT performance. One of these models, the expectancy valence learning (EVL) model, assumes that three processes, namely motivation, memory/learning, and response consistency, are involved in performance on the IGT (24).

The EVL model suggests that deck selection in each trial of the IGT is made based on the expectation of valence, such as a positive or negative feeling for a certain deck or an implicit association between a certain deck and outcomes of gains/losses. This process is called the motivational parameter (25). Expectation of valence is also formed by memory or learning. For example, participants who have deficits of memory or learning cannot use information about gains/losses obtained from earlier trials for selecting decks in later trials. Therefore, the learning parameter reflects how well participants remember experience obtained from earlier trials and apply it to deck selection in later trials. Finally, the response consistency parameter reflects whether participants consistently select decks based on the expectation of valence for each deck or whether they select decks at random (25).

Ahn et al. (26) modified the EVL model and proposed the prospect valence learning (PVL) model. The PVL model separates the EVL model's motivational parameter into feedback sensitivity and loss aversion parameters. The PVL model thus analyzes IGT performance according to four parameters: feedback sensitivity, loss aversion, learning, and response consistency (25). Ahn et al. (26) suggested that the PVL model is better than the EVL model in analyzing IGT performance. For example, they suggested that the PVL model accounts for the gain-loss frequency effects on the formation of expectancy for each deck, which the EVL model cannot explain, since the PVL model uses a non-linear utility function instead of the linear function used by the EVL model (26).

The feedback sensitivity parameter reflects the non-linear relationship between the actual quantities of gains/losses and the prospect valence. A higher feedback sensitivity score means that the participant's subjective evaluation about outcomes of deck selection corresponds more closely to the actual quantities of gains/losses. The loss aversion parameter evaluates the tendency to avoid losses relative to the tendency to seek gains, while the learning parameter measures the formation or modification of preference for each deck based on recent experiences with a particular outcome. Finally, the response consistency parameter reflects the consistency of choice behavior.

The PVL model has been used to analyze the IGT performance in several clinical groups including people with schizophrenia (27) and substance abuse problems (28). It has proven to be a useful tool for revealing the mechanisms underlying decision-making deficits in these clinical groups. For example, Chan et al. (25) compared the IGT performances of patients with AN or bulimia nervosa with those of healthy controls using the PVL model. They found that both the AN and bulimia nervosa groups exhibited significantly impaired performance on the IGT relative to healthy controls. In addition, impaired performance on the IGT was related to memory deficits and sensitivity to gains in patients with AN and bulimia nervosa, respectively.

The investigation of cognitive functions in patients with AN may be affected by several factors such as symptom severity, duration of illness, or comorbidity (21, 29). One way to control these factors is by evaluating individuals at high risk for AN (30), since AN symptoms such as distorted body image (31) and deficits in set-shifting (30) frequently observed in patients with AN are also observed in these individuals. Investigation of high risk group for AN would provide valuable information about the risk factors and prevention programs for AN (32).

To this end, in this study we used the PVL model to analyze IGT results, to investigate decision-making abilities in female college students at high risk for AN. The objectives of the study were to investigate whether female college students at high risk for AN exhibited deficits in decision-making and, if so, to identify the specific decision-making processes underlying impaired IGT performance in these individuals.

## Materials and Methods

### Participants

We administered the Korean version of the Eating Attitude Test-26 (KEAT-26, 33, 34) to 652 female college students. Students who obtained total scores above 22 on the KEAT-26 were included in the high risk for AN group (*n* = 42) and those who obtained average scores were included in the control group (*n* = 43). Garner et al. (33) administered the EAT-26 to AN patients and female college students, and found that total scores above 20 predict the development of AN. Rhee et al. (34) administered the KEAT-26 to Korean women aged over 18 years, and suggested that total scores of above 22 reliably indicate a high-risk of development of AN.

To ensure that none of participants had histories of neurological disorders, mental disorders, or alcohol/drug abuse or addiction, we administered the Structured Clinical Interview for DSM-IV-Non Patient [SCID-NP, (35)]. The Self-Rating Depression Scale [SDS, (36)] and the State-Trait Anxiety Inventory [STAI, (37)] were administered to evaluate depression and anxiety, respectively. Finally, we also administered the Korean version of the Wechsler Adult Intelligence Scale-IV [K-WAIS-IV, (38)]. All participants provided their written informed consent after receiving a description of the study. The students were paid for their participation, and this study was approved by the Sungshin Women's University Institutional Review Board (SSWUIRB 2017-069).

### Eating Attitude Test-26 (EAT-26)

Although the EAT-26 (33) was developed to evaluate characteristics of behavior and attitude in patients with AN, it is also useful for the evaluation of eating behaviors in the general population (33). The EAT-26 consists of 11 items concerning anorexia, seven items concerning binging or overeating, and eight items concerning control of eating behavior. Each item is rated on a 6-level likert scale. In this study, we used the Korean version of the Eating Attitude Test-26 [KEAT-26, (34)].

### Iowa Gambling Task (IGT)

The computerized IGT (39) was administered to evaluate decision-making. In this task, four decks are presented on a computer monitor. When a deck is selected, numbers representing gains and losses are presented on the upper part of the monitor. Gains occur whenever a deck is selected, but the losses occur in certain ratios. The quantities and frequencies of gain/loss are different for each deck (Table 1). Decks A and B deliver large immediate gains but more losses than gains in the long run, whereas decks C and D deliver small immediate gains but more gains than losses overall. No instruction about which decks are advantageous or disadvantageous is given to participants. Instead they are instructed to gain as much as possible before the completion of the task.

**Table 1:** Magnitude and frequency of gain and loss in each block of IGT.

**IGT**	**Deck A**	**Deck B**	**Deck C**	**Deck D**
Mean gain	+$100	+$100	+$50	+$50
Mean loss	–$250	–$1,250	–$50	–$250
Loss probability	5 every 10 trials	1 every 10 trials	5 every 10 trials	1 every 10 trials
Expected value	–$250	–$250	+$250	+$250

*IGT, Iowa gambling task*.

The IGT consists of 120 trials, including 20 practice trials, from which the total net score and block net scores are calculated. The total net score is calculated by subtracting the frequency with which disadvantageous decks are selected from the frequency of selecting advantageous decks [(C+D)–(A+B)]. The 100 experimental trials are divided into five blocks of 20 trials each, and the block net scores are calculated for each block in the same way as the total net score.

### Prospect Valence Learning (PVL) Model

The PVL model suggests that deck selection in each trial of the IGT is based on expectancy valence, which is formed by the magnitude of gains/losses, loss aversion and feedback sensitivity.

$$u\text{​}\left(t\right)=\{\begin{array}{c} x{\left(t\right)}^{a}\;\;\;\;\;\;\;\;\;\;\;if\;x(t)\geq 0\\ -\text{λ}|x{\left(t\right)|}^{a}\;\;\;\;\;\;\;if\;x(t)<0. \end{array}$$

This equation explains how a participant's subjective expectancy valence [*u*(*t*)] is formed, and *x*(*t*) means the net gain on the *tth* trial, i.e., sum of subtracting losses from gains on the *tth* trial. *x*(*t*)^*a*^ determines the feedback sensitivity, i.e., the non-linear relation between actual net gain and expectancy valence, and has 0–1 value. If the value approaches 1, individual's expectancy valence is affected sensitively by the changes of actual amount of gains/losses, whereas if the value approaches 0, the expectancy valence is not affected by actual amount of gains/losses. When the net gain is below 0, motivation to avoid the losses occurs, and this motivation also affects the formation of a participant's subjective expectancy valence along with feedback sensitivity. In the above equation, λ is the loss aversion parameter, i.e., tendency to respond sensitively to losses relative to gains, which has 0–5 values. If λ = 0, participants do not consider the loss at all in the formation of expectancy valence, whereas if λ = 1 participants consider the losses and gains equally. If λ > 1, participants tend to focus more on the losses than gains in the formation of expectancy valence.

PVL model suggests that expectancy valence is also affected by learning, i.e., experience of gains/losses on earlier trials, and the learning parameter is calculated by the following equation.

$$\begin{array}{l} E_{j}\left(t\right)=A\cdot E_{j}\left(t-1\right)+\delta_{j}\left(t\right)\cdot u\left(t\right) \end{array}$$

In the equation, learning parameter A determines how the earlier expectancy valence on the *j* card [*E*_*j*_(*t* − 1)] is considered in the formation of expectancy valence on the *j* card on current trial. δ_*j*_(*t*) is a pacifier variable and coded 1 if *j* card is selected on *tth* trial, whereas 0 if not selected. The learning parameter (0 < A < 1) indexes the learning rate, and the value approaches 1, the expectancy valence formed from earlier experience affects the deck selection on current trial.

The response consistency parameter of the PVL model is calculated by the following equation.

$$\begin{array}{l} Pr\left[D\left(t+1\right)=j\right]=\frac{e^{\theta \left(t\right)\cdot E_{j}\left(t\right)}}{\sum_{k=1}^{4}e^{\theta \left(t\right)\cdot E_{k}\left(t\right)}} \end{array}$$

On the IGT performance, participants form the expectancy valence for each deck through the evaluation of decks, and during earlier trials they explore the decks to assure the expectancy valence for each deck. Later they consistently select the decks having high expectancy valence (26). In the equation, *Pr*[*D*(*t* + 1) = *j*] reflects the probability of choosing *j* card on other trials, and θ*(t)* reflects the degree of selecting decks based on the expectancy valence. Since the PVL model applies a trial-dependent choice rule, [θ*(t)* = 3^c^-1], θ increases or decreases as trials progress. The response consistency parameter (*c*) has 0–5 values, high values reflect consistent deck selection based on expectancy, whereas low values reflect random deck selection.

### Statistical Analysis

The demographic characteristics of the high risk for AN and control groups were analyzed using independent *t*-tests. Total net IGT scores were analyzed using univariate analysis of covariance (ANCOVA), with SDS and STAI as covariates. Block net scores were analyzed with a mixed-design ANCOVA with block as a within-subject factor, group as a between–subject factor, and SDS and STAI as covariates. The relationships between performance on the IGT and severity of AN symptoms were analyzed using the Pearson product-moment correlation.

A Markov chain Monte Carlo sampling scheme in OpenBugs and BRugs [which provides an R interface for OpenBugs, (40)] was used to estimate PVL parameters. After a 500-sample burn-in with three chains, 1,000 samples were drawn and the estimated parameters were analyzed with a Mann–Whitney *U*-test. Relations between PVL parameters and severity of AN symptoms and between PVL parameters and IGT performance were analyzed by bootstrapped Pearson product correlation.

## Results

### Demographic Characteristics

The high risk for AN and control groups did not differ in terms of age, *t*_(83)_ = 1.61, *p* = 0.112, educational level, *t*_(83)_ = 0.52, *p* = 0.603, body mass index (BMI, kg/m^2^), *t*_(83)_ = −0.88, *p* = 0.381, or IQ, *t*_(83)_ = −0.90, *p* = 0.370. However, the two groups differed significantly in SDS, *t*_(83)_ = 4.68, *p* < 0.001, state anxiety as measured by STAI, *t*_(83)_ = 2.18, *p* < 0.05, trait anxiety as measured by STAI, *t*_(83)_ = 5.68, *p* < 0.001, and KEAT-26 score, *t*_(83)_ = 21.78, *p* < 0.001. The high risk for AN group exhibited significantly higher scores on these scales than the control group did. The mean scores of demographic characteristics, depression, anxiety, and eating disorder tests for the high risk for AN and control groups are presented in Table 2.

**Table 2:** Demographic characteristics of high risk for anorexia nervosa (AN) and control groups.

**Demographic variables**	**High risk for AN (*n* = 42)**		**Control (*n* = 43)**		***t*-value**	***p*-value**
	**Mean (SD)**	**[95% CI]**	**Mean (SD)**	**[95% CI]**		
Age (years)	21.43 (2.28)	[20.79, 22.10]	20.70 (1.90)	[20.19, 2.26]	1.606	0.112
Education (year)	14.86 (1.54)	[14.40, 15.35]	14.67 (1.69)	[14.23, 15.15]	0.521	0.603
BMI	19.90 (1.97)	[19.36, 20.53]	20.27 (1.87)	[19.70, 20.87]	−0.881	0.381
IQ	103.21 (9.86)	[100.15, 106.10]	105.14 (9.82)	[102.22, 108.13]	−0.902	0.370
SDS	47.74 (8.74)	[45.14, 50.56]	40.14 (6.00)	[38.41, 41.98]	4.682	0.000
STAI (state)	56.02 (11.23)	[52.61, 59.39]	49.98 (14.12)	[45.84, 54.00]	2.182	0.032
STAI (trait)	51.81 (11.86)	[48.14, 55.23]	38.35 (9.93)	[35.21, 41.14]	5.679	0.000
KEAT-26	31.60 (8.18)	[29.32, 34.16]	3.12 (2.56)	[2.36, 3.93]	21.782	0.000

*BMI, Body mass index; IQ, Intelligence quotient; SDS, Self-rating depression scale; STAI, State-trait anxiety inventory; KEAT-26, Korean version of eating attitude test-26*.

### IGT Performance

**Figure 1:**
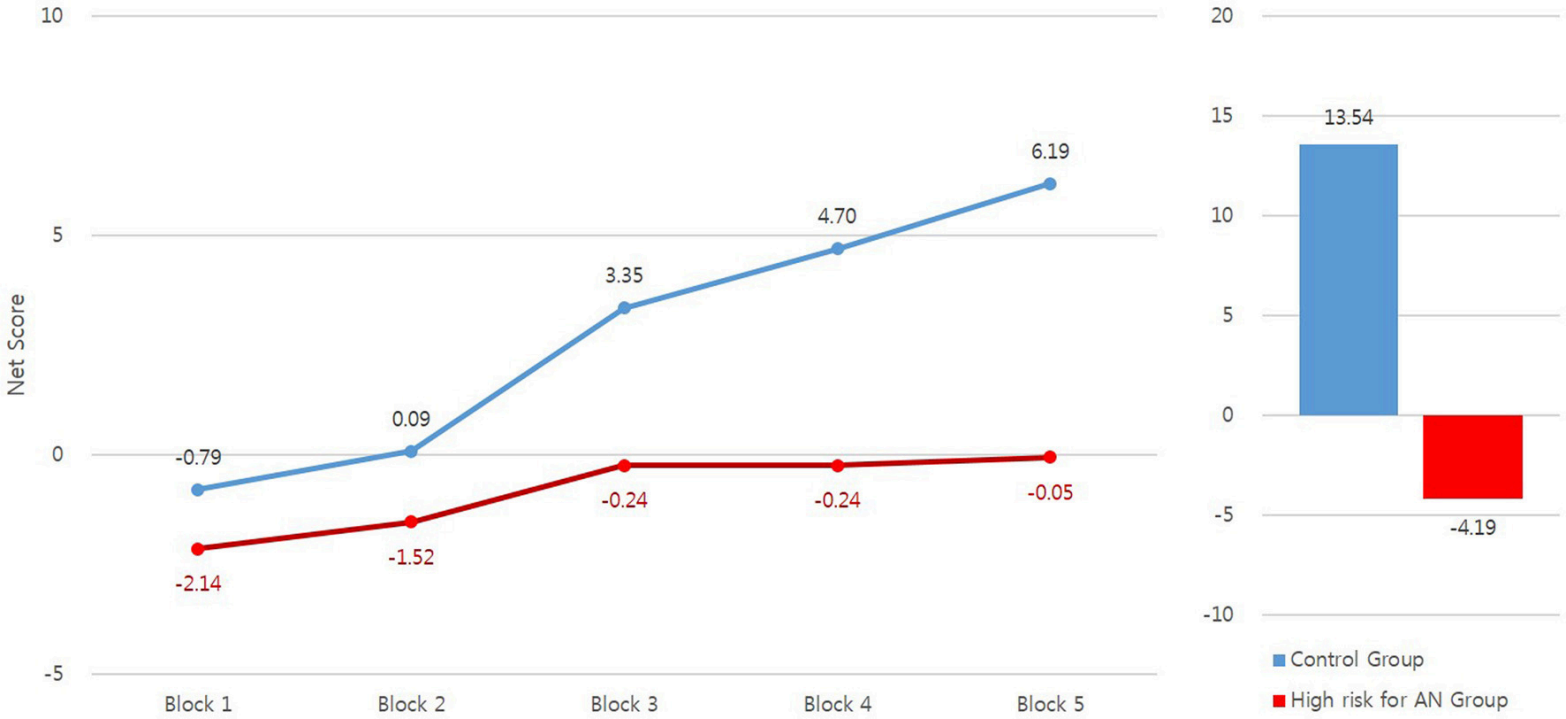
The mean total net scores and block net scores of the IGT in high risk for anorexia nervosa (AN) and control groups.

The total net scores of the high risk for AN and control groups differed significantly, *F*_(1, 80)_ = 9.24, *p* < 0.01, $\eta_{p}^{2}$ = 0.104, with the high risk for AN group exhibiting significantly lower total net scores than the control group. We observed an interaction effect of group X block on block net scores, *F*_(4, 320)_ = 2.69, *p* < 0.05, $\eta_{p}^{2}$ = 0.033. The block net scores of the two groups for each block and the performance of each group over the five blocks were analyzed with univariate ANCOVA and repeated–measures ANOVA, respectively. Bonferroni corrections were used to reduce type 1 errors. The high risk for AN group exhibited significantly lower block net scores than the control group in the third block, *F*_(1, 80)_ = 6.05, *p* < 0.05, $\eta_{p}^{2}$ = 0.070, fourth block, *F*_(1, 80)_ = 7.67, *p* < 0.01, $\eta_{p}^{2}$ = 0.088, and fifth block, *F*_(1, 80)_ = 9.54, *p* < 0.01, $\eta_{p}^{2}$ = 0.107. However, the two groups did not differ in the first, *F*_(1, 80)_ = 1.36, *p* = 0.247, $\eta_{p}^{2}$ = 0.017, or second block, *F*_(1, 80)_ = 1.35, *p* = 0.248, $\eta_{p}^{2}$ = 0.017. In addition, the block net scores of the high risk for AN group did not differ across the five blocks, *F*_(4, 164)_ = 1.64, *p* = 0.176, $\eta_{p}^{2}$ = 0.038, whereas those of the control group increased as the blocks progressed, *F*_(4, 168)_ = 12.26, *p* < 0.001, $\eta_{p}^{2}$ = 0.226. The mean total net scores and block net scores of the high risk for AN and control groups are presented in Figure 1.

**Figure 2:**
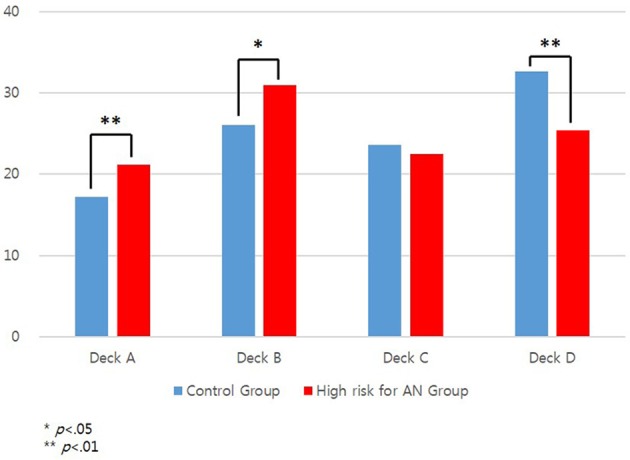
The mean numbers of deck selection of the IGT in high risk for anorexia nervosa (AN) and control groups.

Analysis of deck selection showed that the high risk for AN group selected A, *t*_(83)_ = 2.71, *p* < 0.01, and B decks, *t*_(83)_ = 2.51, *p* < 0.05, more frequently than did the control group, and D deck, *t*_(83)_ = −3.08, *p* < 0.01, less frequently than did the control group. The mean frequencies of deck selection by the two groups are presented in Figure 2.

### Correlations Between IGT Performance and Severity of AN

There was a significant negative correlation between IGT total net score and KEAT-26 score, *r*(42) = −0.24, *p* < 0.05, with subjects in the high risk for AN group who displayed more AN symptoms receiving lower IGT total net scores. However, this correlation was not observed in the control group.

### PVL Model Parameters

The high risk for AN group received significantly lower scores than the control group with regard to the learning (*U* = 579.00, *p* < 0.01) and response consistency (*U* = 633.00, *p* < 0.05) parameters. The two groups did not differ on the feedback sensitivity (*U* = 808.00, *p* = 0.404) and loss aversion (*U* = 738.50, *p* = 0.148) parameters.

There were significant negative correlations between IGT total net scores and the learning, *r*(42) = −0.36, *p* < 0.05 and response consistency parameters, *r*(42) = −0.35, *p* < 0.05 with individuals in the high risk for AN group, whereas there were significant positive correlations between IGT total net scores and the learning, *r*(43) = 0.53, *p* < 0.001, and response consistency parameters, *r*(43) = 0.35, *p* < 0.05, in the control group. There were no significant correlations between PVL model parameters and severity of AN symptoms. The mean values of the PVL model parameters for the high risk for AN and control groups are presented in Table 3.

**Table 3:** The mean values of PVL model parameters of high risk for anorexia nervosa (AN) and control.

**PVL parameters**	**High risk for AN (*n* = 42)**	**Control (*n* = 43)**	***U*-value**	***p*-value**
Feedback sensitivity	0.249 (0.084)	0.268 (0.092)	808.00	0.404
Loss aversion	0.355 (0.390)	0.523 (0.639)	738.50	0.148
Learning	0.377 (0.115)	0.464 (0.188)	579.00	0.004
Response consistency	0.393 (0.418)	0.634 (0.478)	633.00	0.018

*PVL, Prospect Valence Learning; ( ), standard deviation*.

## Discussion

This study investigated deficits of decision-making in female college students at high risk for AN using the IGT and PVL model. Analysis of demographic characteristics showed that the high risk for AN group exhibited significantly higher levels of depression and anxiety than did the control group. These results are consistent with those of previous studies that found depression and anxiety disorders to be comorbid with AN (41, 42).

The total net IGT scores of the high risk for AN group were significantly lower than those of the control group, indicating that individuals at high risk for AN experience deficits in decision-making ability. Although these results are consistent with the findings of some studies (13, 21), other studies have produced contradictory results (43, 44). For example, Guillaume et al. (44) did not find significant differences in IGT performance between patients with AN and healthy controls after controlling for depression and medication, leading them to conclude that variables such as depression or medication contributed to impaired IGT performance in patients with AN. However, the present results, i.e., the finding that IGT performance is impaired in individuals at high risk for AN after controlling for depression and anxiety, indicate that the deficits in decision-making are trait characteristics which are not affected by depression or anxiety, and preexist the development of AN.

The high risk for AN group exhibited significantly lower block net scores in the third, fourth and fifth block of the IGT than the control group did. The controls learned which decks were advantageous or disadvantageous and selected advantageous decks more frequently than disadvantageous ones as the trials progressed, whereas this learning did not occur in the high risk for AN group. To perform the IGT successfully, participants must learn the contingencies of gains/losses for each deck. Bechara et al. (45) stated that healthy participants switch from one deck to another through trial and error at the outset, but that as trials progress (after about 50 trials, in the third block of the IGT) participants begin to recognize that decks A and B are disadvantageous and their preference becomes biased toward decks with higher net gain. In other words, healthy participants develop a preference for advantageous decks. The present results showed that this development of preference does not occur in the high risk for AN group. These results suggest that the high risk for AN group may have limited behavioral or mental flexibility, since difficulties in set-shifting are frequently observed in patients with AN (21) or individuals at high risk for AN (30), and significant associations between performances on the IGT and the reversal-learning task are observed (46).

The high risk for AN group selected cards from decks A and B more frequently and from deck D less frequently than did the control group. Decks A and B deliver larger gains but also larger losses than decks C and D (see Table 1), leading to lower net gains in the long run. The high frequency with which the high risk for AN group in this study selected decks A and B indicates that these individuals prefer immediate large gains despite greater losses in the long run. Additionally, the high risk for AN group selected deck D significantly less frequently than the control group did, while the two groups did not differ in the frequency with which they selected deck C. Although decks C and D are both advantageous, deck D delivers larger (–$250 vs. –$50) but less frequent (10 vs. 50%) losses than does deck C. Healthy participants tend to select the deck that delivers less frequent losses (47). However, individuals in the high risk for AN group avoided larger losses and were unable to simultaneously consider both the magnitude and frequency of gains/losses when selecting decks. Furthermore, results of deck selection indicate that the high risk for AN group selected decks based on immediate cues, such as large gains or losses, rather than long-term outcome.

There was a significant negative correlation between IGT total net score and AN symptoms measured by the KEAT-26 in the high risk for AN group. The orbitofrontal cortex is involved in performance on the IGT (22). Reduced orbitofrontal volume is observed in patients with AN and a relationship between reduced orbitofrontal volume and IGT performance has also been reported (20). Extrapolating from these data, the present results indicate that individuals at high risk for AN may have structural or functional dysfunctions of the orbitofrontal cortex.

Application of the PVL model revealed that poor contingency learning was related to poor IGT performance in individuals at high risk for AN. The high risk for AN group exhibited significantly lower values for the learning parameter of the PVL model than the control group, and this result is consistent with previous observation of AN patients (25). The learning parameter measures the formation or modification of preference for each deck based on recent experiences with a particular outcome (25, 26). Our results indicate that individuals at high risk for AN could not memorize or incorporate experiences from earlier trials into expectancies for subsequent trials, or modify preferences formed during earlier trials. In other words, individuals at high risk for AN may have difficulties with memory (48), working memory (49), or set-shifting (13, 21, 50).

The high risk for AN group also exhibited lower response consistency values than the control group. The response consistency parameter reflects the degree of consistency between deck selections and the expected outcomes associated with each deck (24, 25). In the present study, the high risk for AN group selected cards randomly throughout the trials without learning which decks were advantageous or disadvantageous, whereas the control group consistently selected cards from advantageous decks as trials progressed (51). Application of the PVL model revealed that the high risk for AN group had significantly lower values of the learning and response consistency parameters. These results indicate that individuals at high risk for AN have deficits in decision-making ability, possibly due to failures to convert experiences from previous trials into expectancies about options in subsequent trials.

This study has several limitations that should be addressed in future research. First, inclusion of only a small number of female participants in this study limits the generalizability of our findings. Second, menstrual cycle, which is known to affect decision-making ability in women (52), was not controlled in the present study. Third, as the PVL model is based on behavioral data from the IGT, its ability to reveal the mechanisms leading to poor IGT performance in individuals at high risk for AN is limited. Therefore, future studies should employ neuroimaging techniques to understand these mechanisms more fully. Fourth, performance on the IGT may be associated with performance on neuropsychological tests evaluating memory or set-shifting. Future studies should administer both the IGT and neuropsychological tests to provide a better understanding of the association between decision-making ability and other neuropsychological functions. Finally, subjects in the high risk for AN group could not be classified by AN subtype in the present study. Since characteristics such as impulsivity or perfectionism differ according to subtypes of AN (53), studies that classify high risk for AN group into subtypes would provide valuable information about the risk factors for AN.

In conclusion, individuals in the high risk for AN group had significantly lower total net scores and block net scores in the third, fourth, and fifth blocks of the IGT. They were more likely than controls to select cards from the disadvantageous A and B decks and less likely to select them from the advantageous D deck. Additionally, the block net scores of the control group increased as trials progressed, whereas the block net scores of the high risk for AN group did not change over the course of the IGT. Application of the PVL model showed that the high risk for AN group had significantly lower values for the learning and response consistency parameters. These results indicate that individuals at high risk for AN have deficits in decision-making, possibly due to failure to incorporate the experiences of outcomes on previous trials into card selection in subsequent trials.

## Author Contributions

M-SK designed the study and revised the manuscript. EN collected the data and wrote the first draft. BK analyzed the data.

### Conflict of Interest Statement

The authors declare that the research was conducted in the absence of any commercial or financial relationships that could be construed as a potential conflict of interest.
